# Method of precise optical crystal alignment by tilting the diamond anvil cell

**DOI:** 10.1107/S1600576724012500

**Published:** 2025-02-01

**Authors:** Eduard B. Rusanov, Michael D. Wörle, Maksym V. Kovalenko

**Affiliations:** a Department of Chemistry and Applied Biosciences ETH Zürich Vladimir-Prelog-Weg 1-5/10 Zürich8093 Switzerland; b Department of Physicochemical Investigation Institute of Organic Chemistry at National Academy of Sciences of Ukraine 5 Akademik Kukhar Str. Kyiv02660 Ukraine; HPSTAR and Harbin Institute of Technology, People’s Republic of China

**Keywords:** optical alignment method, diamond anvil cells, DACs, high-pressure crystallography, Snell’s law

## Abstract

A new, accurate method for fast and precise optical alignment of crystals in a diamond anvil cell (DAC) on a diffractometer has been developed. It enables highly accurate crystal alignment within instruments with a Boehler–Almax DAC design, achieving precision better than 0.02 mm easily. Other advantages of this method are simplicity, speed and instant visual feedback when aligning the crystal. This method employs Snell’s law, which relates the angles of incidence and refraction of light passing through different media to estimate the crystal position within the DAC by measuring the apparent transverse dis­placement of the crystal image at various viewing angles after rotating the DAC by 180°. This information allows for fine-tuning of the crystal alignment within the DAC, ensuring optimal conditions for high-pressure diffraction experiments.

## Abstract 2

A novel method for precise crystal alignment in a diamond anvil cell has been developed. This method leverages Snell’s law providing immediate visual feedback for alignment and offering exceptional accuracy (<0.02 mm) while being simple and fast, facilitating high-pressure diffraction experiments.

## 1. Introduction

Accurate crystal centering is essential for ensuring the reliability and precision of diffraction experiments. The positions of diffracted beams and even their intensities are directly influenced by the quality of the crystal alignment on the diffractometer (Ito *et al.*, 2019 ▸). For diffraction experiments, a single crystal should be precisely positioned at the intersection of the diffractometer axes. Therefore, accurate crystal centering is a fundamental prerequisite for any such study. The standard optical method for crystal centering usually involves crystal rotation by 0, 90, 180 and 270° along the phi axis and is commonly used to center the crystal in standard diffraction experiments. Due to the limited viewing direction imposed by the supporting structure of a diamond anvil cell (DAC) (Merrill & Bassett, 1974 ▸), it is not feasible to achieve precise crystal alignment using the traditional optical methods employed in standard diffraction experiments. There are three primary methods for crystal centering in DACs: optical by focusing the microscope (Budzianowski & Katrusiak, 2004 ▸; Dawson *et al.*, 2004 ▸), gasket shadow (Budzianowski & Katrusiak, 2004 ▸; Kunz *et al.*, 2005 ▸) and diffractometric alignment. The diffractometric alignment method was first introduced by Hamilton (1974 ▸), demonstrating the ability to determine the sample position relative to the rotation center by analyzing single-crystal reflection angles. This method, along with its variations adapted for DACs by King & Finger (1979 ▸), was further generalized by Dera & Katrusiak (1999 ▸).

**Figure 1:**
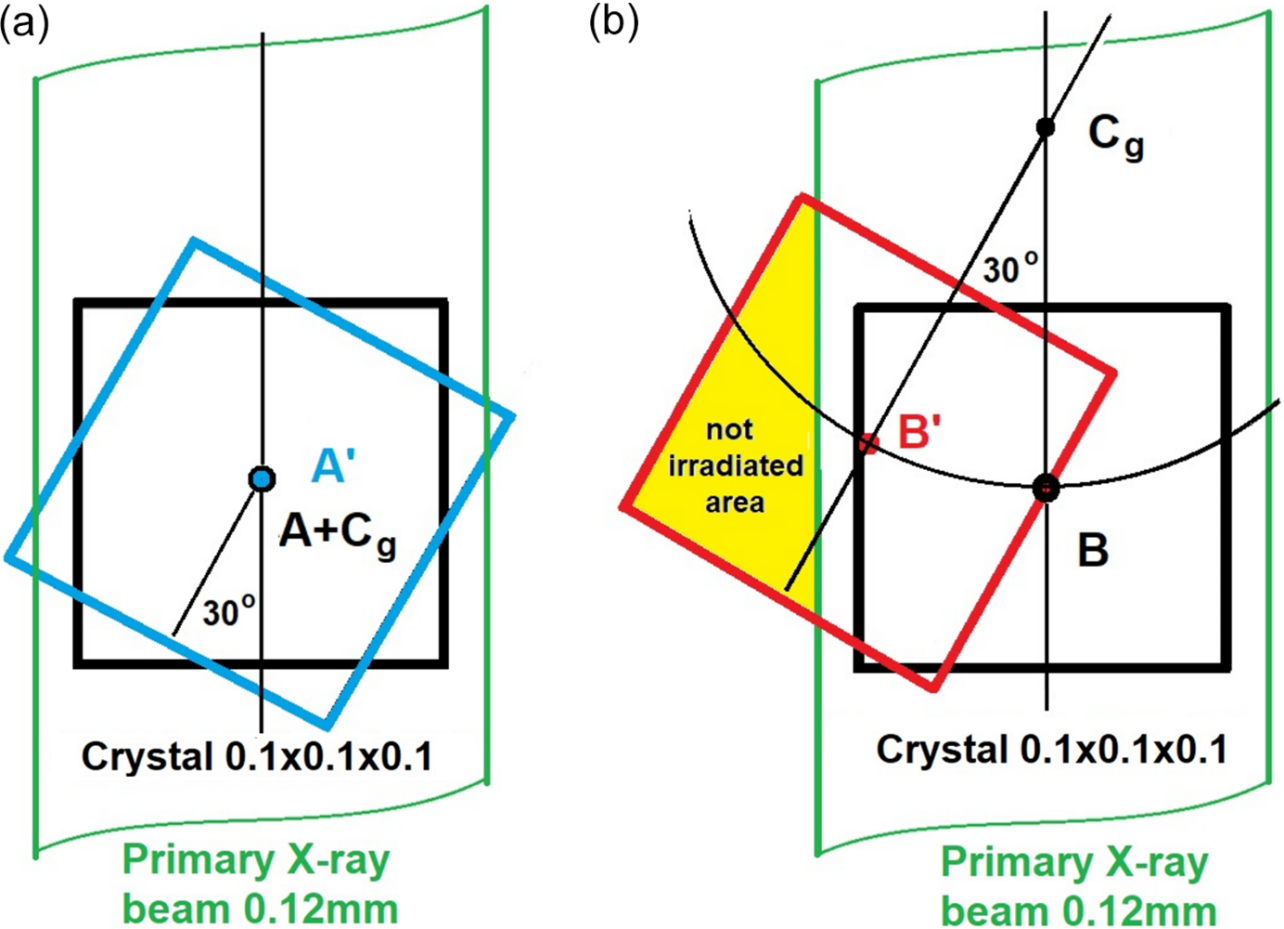
(*a*) Well centered crystal in a primary beam. The crystal center (A) coincides with the goniometer center (C_g_). Rotating the DAC with a crystal by 30° (marked in blue) maintains this alignment, with the crystal center (A′) remaining at (C_g_). (*b*) The crystal center (B) is offset from the goniometer center (C_g_) by 0.1 mm in the direction of the microscope. After rotating the DAC by 30°, the crystal center (B′) is in a significantly different position (marked in red). In this case, a portion of the crystal moves outside the primary beam, reducing the diffracting crystal volume and power.

The effectiveness of the optical centering method is limited by the DAC design, which only allows crystal observation at around 0 and 180° orientations. In optical centering, the DAC must be carefully aligned so that its window is strictly perpendicular to the optical axis of the microscope when the diffractometer circles are at the centering positions. This position is then used to collect diffraction data from the crystal in a diffraction experiment. At the same time, the crystal must be adjusted so that the microscope is focused on the crystal in both positions. This alignment step is crucial for successful crystal centering as well as subsequent data collection and absorption correction (Angel *et al.*, 2000 ▸). Although optical alignment by focusing is a straightforward method, its accuracy of approximately 0.1 mm in the direction of the microscope is insufficient for precise positioning of small crystals when using microfocus X-ray or synchrotron sources with small beam sizes (<0.1–0.12 mm). Such misalignment of the crystal in the direction of the microscope or detector (schematically depicted in Fig. 1 ▸) can significantly impact diffraction data quality. When a crystal is offset by a small amount (*e.g.* 0.1 mm) from the goniometer center, even a 20 or 30° DAC rotation during the data collection can cause partial crystal irradiation, distorting measured reflection amplitudes and compromising *R*_int_/*R*_σ_ values, refinement parameters and the accuracy of the diffraction experiment in general. Other methods offer a more precise degree of centering but are also more complex and time consuming. The gasket shadow method based on X-ray transmission through the DAC provides improved accuracy but is limited to X-ray beams larger than the gasket hole, so in many cases, it is incompatible with microfocus X-ray or synchrotron sources due to their smaller beam sizes. The gasket shadow method centers on the gasket hole rather than the crystal itself, assuming the crystal is precisely positioned at the center of the hole. However, this assumption may not always hold true, especially at high pressures where non-cylindrical hole shapes can compromise the accuracy of the method. Additionally, precise crystal positioning along the microscope axis is challenging due to the placement of the crystal on one of the DACs when the distance between anvils significantly exceeds the crystal size, as is the case at relatively low pressure. This method was later adapted and applied by Smith & Desgreniers (2009 ▸) to centering samples in a DAC using the microfocus radiation source. It is based on a transmission profile obtained by scanning a sample in a DAC across a collimated X-ray beam, and can then be used to monitor the sample displacement caused by rotation. The diffractometric crystal centering provides the highest accuracy, achieving approximately 0.02 mm precision in all directions (Dera & Katrusiak, 1999 ▸). This iterative method, relying on determining the displacement by least-squares fit from the Friedel pairs and equivalent reflections located at different ψ azimuths, offers superior results compared with the optical method by focusing the microscope but demands more time. However, it lacks the immediate visual feedback of optical alignment. Although optical and gasket shadow methods offer practical approaches, diffractometric centering is essential for precise experiments with small crystals and microfocus X-ray or synchrotron sources. Although the latter method of crystal alignment in a DAC has long been widely used in high-pressure crystallography, it is known to be time consuming.

For an extended period, it was believed that precise crystal alignment within a DAC using the optical method was partially or entirely ineffective. This perception stemmed from the challenges posed by the high refractive index of diamonds, which can distort the optical path length as the DAC rotates. One contributing factor to the perceived limitations of optical alignment in early DACs was the use of beryllium plates with a pinhole, which were employed as holders of the DACs. Nowadays, researchers increasingly utilize Boehler–Almax DAC design, which provides optical access to the sample within the chamber at a wide range of angles (>35–40°), enabling various measurements including the application of spectroscopic techniques. It was also known that, because of the very high optical refractive index of diamond (>2.4), even a small DAC misalignment of 2° in φ at the centering positions results in a transverse displacement of the crystal image by 0.03 mm. So, the initial position of the DAC strongly perpendicular to the axis of the microscope or X-ray primary beam can be adjusted in φ with an accuracy better than 0.5° (Angel *et al.*, 2000 ▸). Leveraging the high refractive index of diamond, we proposed a novel method for precise crystal centering in a Boehler–Almax DAC design. Since Snell’s law is used to relate the angles of incidence and refraction of light passing through an interface between two media, its direct application to estimating the position of a crystal in a DAC seems reasonable. Herein we present an improved optical crystal alignment method using DAC tilt.

## 2. Theoretical background of the method

This method employs Snell’s law and can be used to estimate the position of the crystal inside the DAC in the direction of the microscope/camera. When the viewing angle of the crystal differs from the perpendicular to the DAC window, the difference between the apparent displacement of the crystal image depends on the angle of DAC tilt (in a general case, the φ angle on the diffractometer or the angle of incidence in optics) and the position of the crystal inside the DAC. Knowing the viewing angles (φ) of the crystal and the image offset, we can estimate the crystal position inside the DAC for alignment. Using Snell’s law (*n*_1_sinθ_1_ = *n*_2_sinθ_2_, where *n*_1_ and *n*_2_ are refraction coefficients and θ_1_ and θ_2_ are the refraction angles for the two media), we calculated the angles of transmission of light from the crystal through the air/DAC/pressure transmitting medium (PTM). In our calculations, we used a DAC tilt angle of 14° from the ideal position when the DAC window and anvils are strictly perpendicular to the optical axis of the microscope camera. The main reasons for using this angle are the certain positions of the φ axis (14 and 194°) for centering the crystal on a Rigaku diffractometer and fairly large sine values for the angles of incidence and refraction in various media, including the diamond with high refractive index. This enhances the precision of crystal position determination. However, any other reasonable tilt angle values can be used. The following typical conditions were chosen as a starting point for calculations: 0.1 mm crystal size in all directions, a PTM that is a mixture of the pentanes with *n* = 1.36, 0.17 mm gasket thickness and two DACs with the same dimensions whose surfaces are strictly parallel to each other.

**Figure 2:**
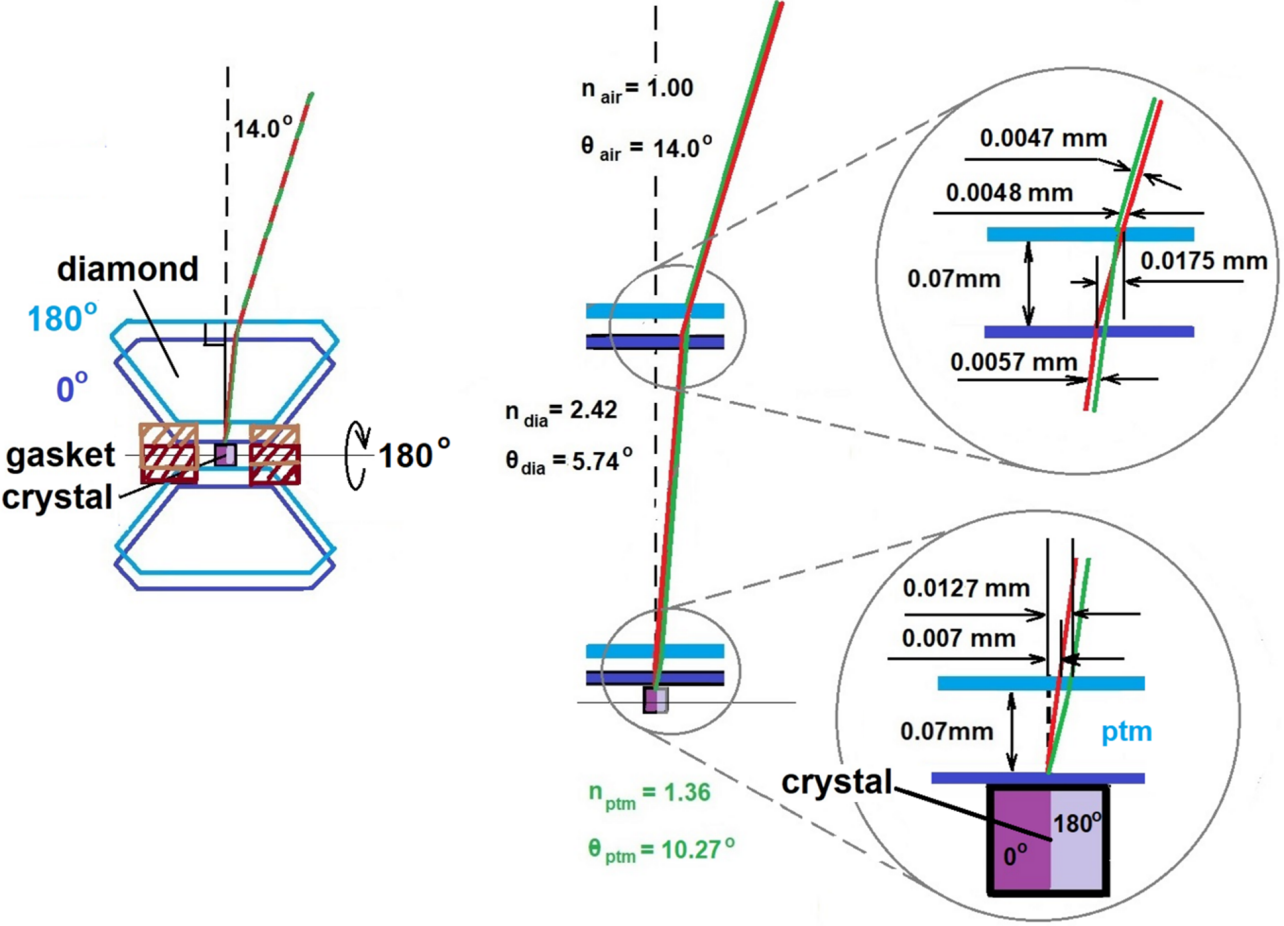
Calculated refraction and light paths within a well centered crystal in the DAC at a 14° angle of incidence. The DAC with a crystal represented by the cube is shown in two orientations: 0 and 180° rotation around the horizontal axis. In these two orientations, the DACs are shown in purple and light blue lines, and the corresponding light paths are shown in red and green. The calculations for the 180° rotation incorporate the influence of an additional 0.07 mm PTM layer. After rotation of the DAC by 180°, the red and green light rays almost completely overlap.

Fig. 2 ▸ illustrates the calculated refraction and light paths within a DAC at a 14° angle of incidence. The DAC is shown in two orientations: 0 and 180° rotation around the horizontal axis. The DACs in these orientations are depicted in purple and light blue, and light paths in red and green, respectively. The calculations for the 180° rotation incorporate the influence of an additional 0.07 mm PTM layer. In the initial scenario, the rays pass solely through air and diamond (red line) since the crystal is fixed on the diamond surface. However, a 180° rotation introduces an additional 0.07 mm-thick layer of PTM into the ray path (green line). Calculations using the law of sines applied to a triangle with the generalized formula *a*/sin α = *b*/sin β = *c*/sin γ (where *a*, *b* and *c* are the sides and α, β and γ are the opposite angles of the triangle) indicate that the lateral ray displacement with respect to the direction of the X-ray beam due to the 0.07 mm-thick PTM layer is very small, since the difference between the ray paths in air and PTM is only 0.07 sin(14°)/sin(76°) − 0.07 sin(10.27°)/sin(79.73°) = 0.0175 − 0.0127 = 0.0048 mm. Thus, for a well centered crystal, rays of light entering the DAC from the 0 and 180° positions follow nearly identical paths. Nevertheless, this very small shift is expected to decrease with increasing pressure due to the accompanying reduction in gasket thickness. Although a minor correction can be applied if necessary, it is generally insignificant.

**Figure 3:**
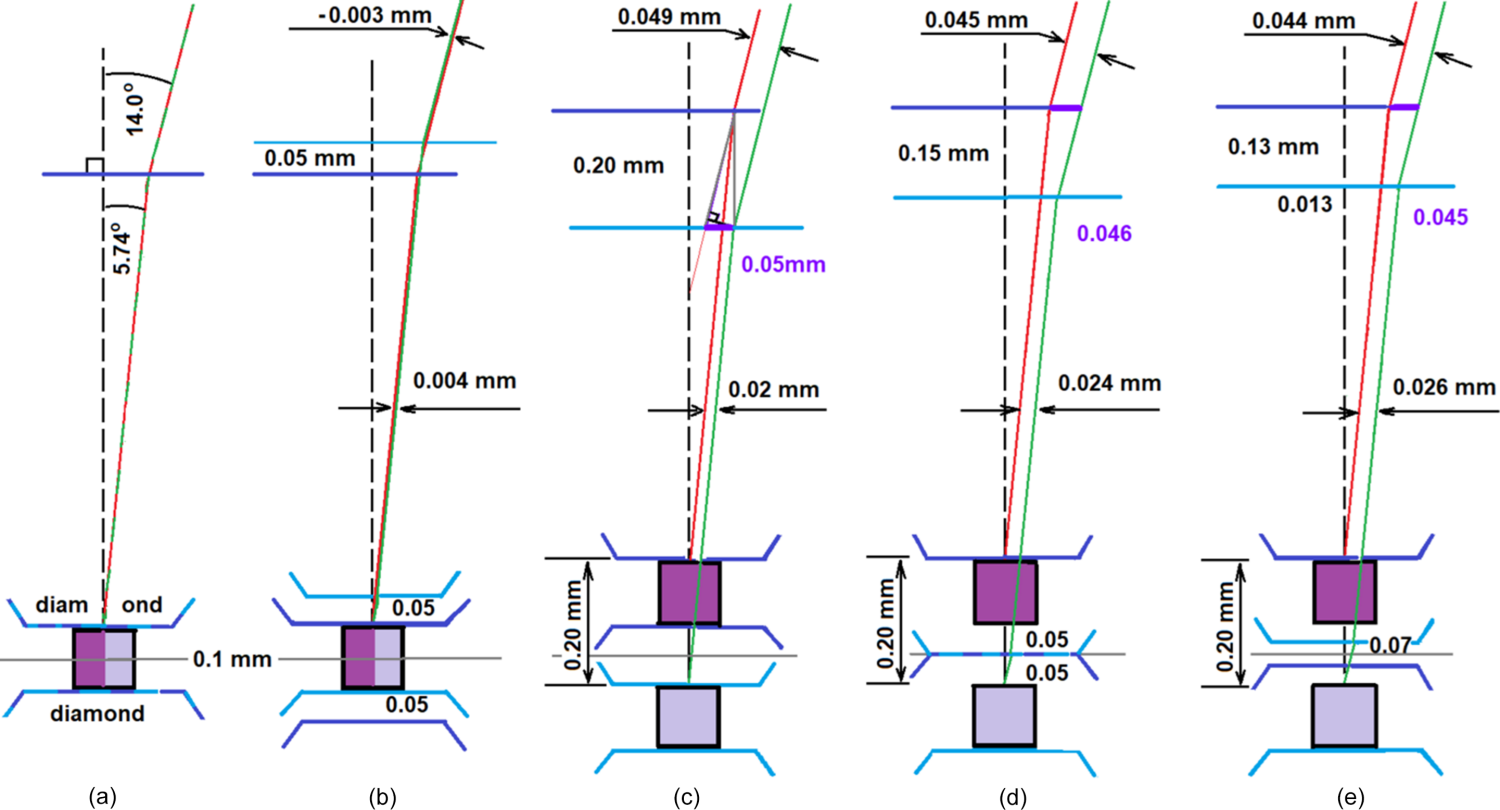
Calculated refraction and light paths within a well centered crystal in the DAC at a 14° angle of incidence (*a*) without an additional PTM layer and (*b*) with a 0.05 mm PTM layer. After rotating the DAC by 180°, the red and green light rays are completely or almost completely overlapped. In scenarios (*c*)–(*e*), the crystal in the DAC is offset from the goniometer center by 0.1 mm. Scenarios (*d*) and (*e*) also introduce an additional PTM layer of 0.05 and 0.07 mm, respectively. After a 180° DAC rotation, the light rays remain parallel in the same type of optical medium. The DAC in two orientations at 0° and after rotating 180° on the vertical axis is shown as purple and light blue lines, respectively, and the corresponding light rays are shown in red and green.

Similar calculations can be performed for various scenarios depicted in Fig. 3 ▸. For a well centered crystal, the rays for the DAC in both 0 and 180° DAC positions (the tilt angle is always 14°) follow identical [Fig. 3 ▸(*a*)] or near-identical paths (with an additional PTM layer of 0.05 mm) [Fig. 3 ▸(*b*)] shown in red and green, respectively, finally resulting in the complete overlap. Calculations indicate that a crystal in the DAC offset in the direction of the microscope from the goniometer center by 0.1 mm without a PTM layer in the DAC leads to a substantial lateral ray displacement of 0.05 mm [Fig. 3 ▸(*c*)]. In the latter case, the difference in the light path within the DAC after a 180° rotation is calculated as 0.2 sin(5.74°)/sin(84.26°) = 0.020 mm. Determining the path difference of rays in air is also not difficult, since it can be easily calculated from a 0.2 mm-high right triangle, and the angles are known to be 14, 90 and 76°, which give a displacement value of 0.05 mm. Calculation of the ray path for a crystal offset from the goniometer center by 0.1 mm with an additional PTM layer of 0.05 mm [Fig. 3 ▸(*d*)] and 0.07 mm [Fig. 3 ▸(*e*)] thickness also leads to a very impressive difference. Thus, the transverse image shift for a crystal that is not well centered is also significant and is easily detectable by the microscope/camera when the DAC is rotated by 180°. Despite the relatively small image shift of approximately 0.045–0.05 mm for a 0.1 mm crystal shift from the center, this method remains highly sensitive and good enough for precise crystal position determination. Given that the microscope/camera on the modern diffractometer can readily detect image displacements of 0.01 mm, the accuracy of this method is expected to be comparable to, if not superior to, precise diffractometric methods.

**Figure 4:**
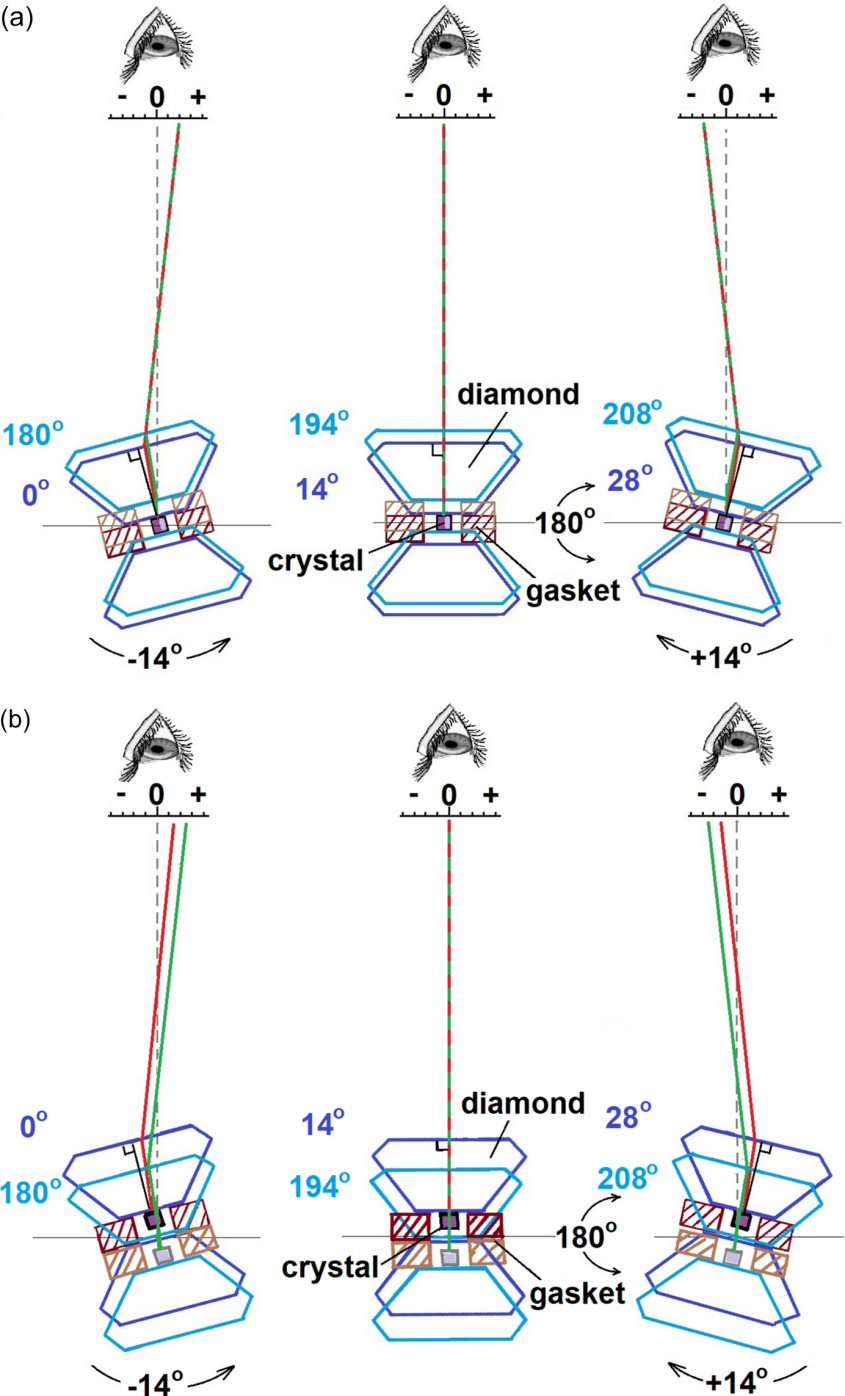
(*a*) For a well centered crystal in the DAC, the apparent offset of the crystal from the center remains unchanged after tilting by 14° in either direction and rotating the DAC by 180°. (*b*) For a crystal slightly offset toward the microscope, tilting the crystal by 14° and rotating the DAC by 180° will result in a noticeable difference in the transverse shift of the crystal images. The DAC in the centered orientation and at a tilt angle of ±14° and after rotation by 180° are represented by purple and light blue outlines; the corresponding light rays are displayed in red and green.

To summarize the above, Fig. 4 ▸ shows a simulation of the light path on a diffractometer for (*a*) a well centered crystal (the light rays overlap completely, resulting in no transverse image displacement after tilting the DAC by ±14° and rotating it 180°) and (*b*) a crystal that is misaligned from the goniometer center in the direction of the microscope. This mis­alignment leads to a clear lateral image displacement when the DAC is tilted by ±14° and rotated 180°. By averaging the apparent crystal position across both positive and negative angular ranges, it is possible to accurately determine the crystal center and its displacement from the goniometer center, particularly for crystals with irregular or challenging shapes. The high refractive index of diamond increases the sensitivity of the method, resulting in crystal alignment accuracy comparable to or better than diffractometric methods (depending on the tilt angles).

## 3. A practical approach for the improved optical crystal centering method by DAC tilt

### 3.1. General remarks

This advanced optical crystal centering method by DAC tilt has been successfully applied in about 100 high-pressure experiments, including those leading to recently published data (Rusanov *et al.*, 2024*a* ▸) obtained on our in-house diffractometer Rigaku Synergy S. A miniature Diacell Tozer DAC supplied by Almax EasyLab with an opening angle of 41° equipped with Boehler–Almax cut diamonds with 0.8 mm culets was used for all experiments in the pressure range up to 3.4 GPa. The gaskets, made of 0.25 mm BeCu alloy or stainless steel foil, were designed to a thickness of 0.17–0.20 mm and a hole diameter of 0.25–0.4 mm. With minimal training, this method enables rapid and precise crystal centering on a diffractometer within a few minutes. With our DAC, due to the high refractive index of diamond compared with air, the camera focus alignment wheel on the diffractometer was turned 1.4–1.5 turns counterclockwise to initiate the DAC alignment process. Subsequent fine adjustments could be made as needed. It is recommended to mark the focus positions with a permanent marker on the camera before adjusting the wheel for high-pressure measurements. This allows for an easy return to the original setup without the need for refocusing during the standard diffraction experiments. The correct focus position of the microscope for a particular DAC type may also be marked in a different color after successful focusing and centering of the crystal in the DAC. These straightforward procedures enable rapid adjustment of the diffractometer and camera focus settings.

### 3.2. The procedure of improved optical centering

An improved method of optical centering consists of the following steps:

(1) The initial rough alignment of the crystal is to ensure that the DAC window is positioned as perpendicular as possible to the optical axis of the microscope and that the crystal is centered within the field of view. This initial alignment process involves adjusting the focus of the microscope as needed, and fine-tuning of the crystal position using the goniometer head sliders in combination with fine manual rotation of the DAC (not the goniometric head itself) around the φ axis is necessary to achieve a stable centered position after a 180° rotation. As this initial alignment process is detailed in the references, and the practical approach is also explained in the Rigaku High Pressure Workshop [https://www.youtube.com/watch?v=pleIvAXtL5o&t=1754s], we will not provide any further explanation for this step.

(2) A fine crystal alignment along the microscope axis is achieved using the developed DAC tilt method. The following section will demonstrate this approach using a Rigaku diffractometer configured for high-pressure experiments (HP MODE). As necessary it can be easily adapted to any other diffractometer since it employs only φ rotation for the centering.

For the Rigaku Synergy S diffractometer, the initial alignment positions of ‘0 Arrow Down’ and ‘180 Arrow UP’ (Step 1) correspond to internal goniometer angles (φ) of 14 and 194°, respectively (see Fig. 4 ▸). To evaluate the method capabilities, we intentionally tested it using small tilt angles of ±6 and ±8°, despite recognizing that the accuracy may be limited at these angles. So, the procedure involves the following steps: (*a*) start initial alignment at φ = 14 or 194°, (*b*) rotate the goniometer by ±6° (to φ = 8, 20, 188, 200°) and (*c*) rotate the goniometer by ±8° (to φ = 6, 22, 186, 202°).

**Figure 5:**
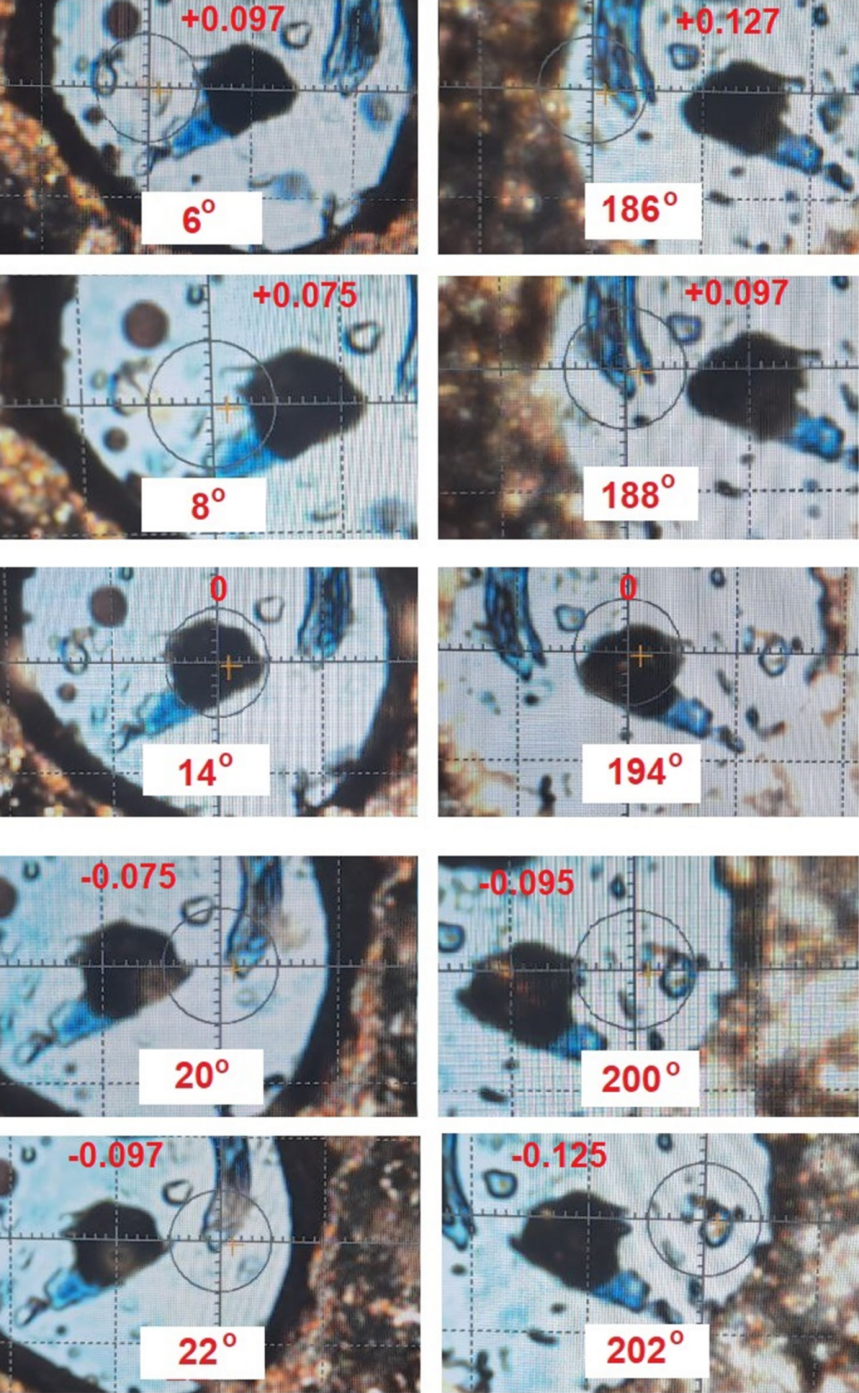
Images of the apparent crystal positions for an initially pre-aligned crystal at φ of 14 and 194° using the standard optical centering method by focusing the microscope. The DAC was subsequently tilted by ±6 and ±8°. After tilting, a slightly offset crystal exhibits a noticeable image shift when the DAC is rotated 180° about the φ axis. The images display the corresponding φ angles on the diffractometer and indicate the displacement of the crystal from the optical microscope target in millimetres. The observed crystal offset and the difference in offset for a poorly centered crystal increases with tilt angle, demonstrating the enhanced sensitivity of the method.

Fig. 5 ▸ shows the apparent crystal position for an initially pre-aligned crystal using the standard optical centering method by focusing the microscope (with φ = 14 and 194°) and after DAC tilting for ±6 and ±8°. The data in Table 1 ▸ summarize the crystal center position values taken from Fig. 5 ▸ after tilting the DAC by φ ±6 and 8°, and additional data at ±10°. Following the theoretical background of the method above, at the point with perfect crystal centering, the apparent deviation of the crystal from the goniometer center should be the same for images obtained in pairs at φ angles such as 6 and 186° (tilt angle −8°), or 22 and 202° (tilt angle +8°). By examining the crystal position at both positive and negative tilt, systematic errors caused by crystal shape imperfections can be minimized, leading to improved centering accuracy. Data analysis reveals a direct correlation between the apparent crystal position deviation from the center and the tilt angle. While the crystal center position for identical angles on opposite sides of the rotation appears similar (Table 1 ▸), comparing points separated by 180° reveals subtle discrepancies indicative of misalignment. By calculating the average offset, we can identify the position of the crystal and correct any misalignment. For instance, at φ angles of 6 and 186° (DAC tilt −8°), the center of the crystal is at +0.097 and +0.127 mm, respectively, indicating significant misalignment when using the standard optical microscope focusing method. A similar misalignment is observed at 22 and 202° since the crystal center at these positions is −0.097 and −0.125 mm. These observations demonstrate that the crystal/DAC assembly should be adjusted to a midpoint position, regardless of the starting position. Adjusting the DAC to minimize these deviations is necessary for accurate centering. Thus if the current φ angle is 6 or 186°, the visible center of the crystal after adjustment would be at (0.097 + 0.127)/2 = 0.112 (0.11) mm from the center of the goniometer for correct alignment. For the second pair of angles (φ = 22 and 202°), the observed crystal deviations (−0.097 and −0.125 mm, respectively) suggest a necessary shift of the DAC to the −0.111 (−0.11) mm position. To correct this offset, fine adjustment of the goniometer head using *only* a slider in the direction of the microscope is necessary. Following such adjustments along the microscope axis, the crystal should appear centered. Applying the law of sines, we calculated that the mean apparent image shift difference of 0.021 mm at a 6° tilt angle (Table 1 ▸) corresponds to a maximum crystal displacement (*d*) from the center as *d* = 1/2 [0.021 sin (84°)/sin (6°)] = 0.100 mm. Similar calculations using 8, 10 and 14° tilt angles yielded comparable values of 0.103, 0.092 and 0.083 mm, respectively, confirming the effectiveness of the method. However, the above values do not consider the impact of the PTM layer on the calculations and the accuracy of determining the crystal displacement decreases at small DAC tilt angles. It is obvious that the high refractive index of diamond increases the sensitivity of the method, resulting in crystal alignment accuracy comparable to or better than diffractometric methods (depending on the tilt angles).

To summarize the above, the complete improved DAC tilt crystal alignment method consists of the following simple steps:

(1) Use the standard optical crystal alignment method by focusing the microscope.

(2) Tilt the DAC to a certain angle, say ±14°, and rotate it 180°.

(3) Determine the displacement of the crystal image between corresponding positions and calculate the necessary correction as an average value.

(4) Move the crystal to the desired position using the corresponding slider of the goniometric head in the direction of the microscope.

(5) Rotate the DAC 180° again to verify the alignment as described in steps (2)–(4) and correct the crystal position if necessary.

### 3.3. Examples of application of the improved optical centering method

**Figure 6:**
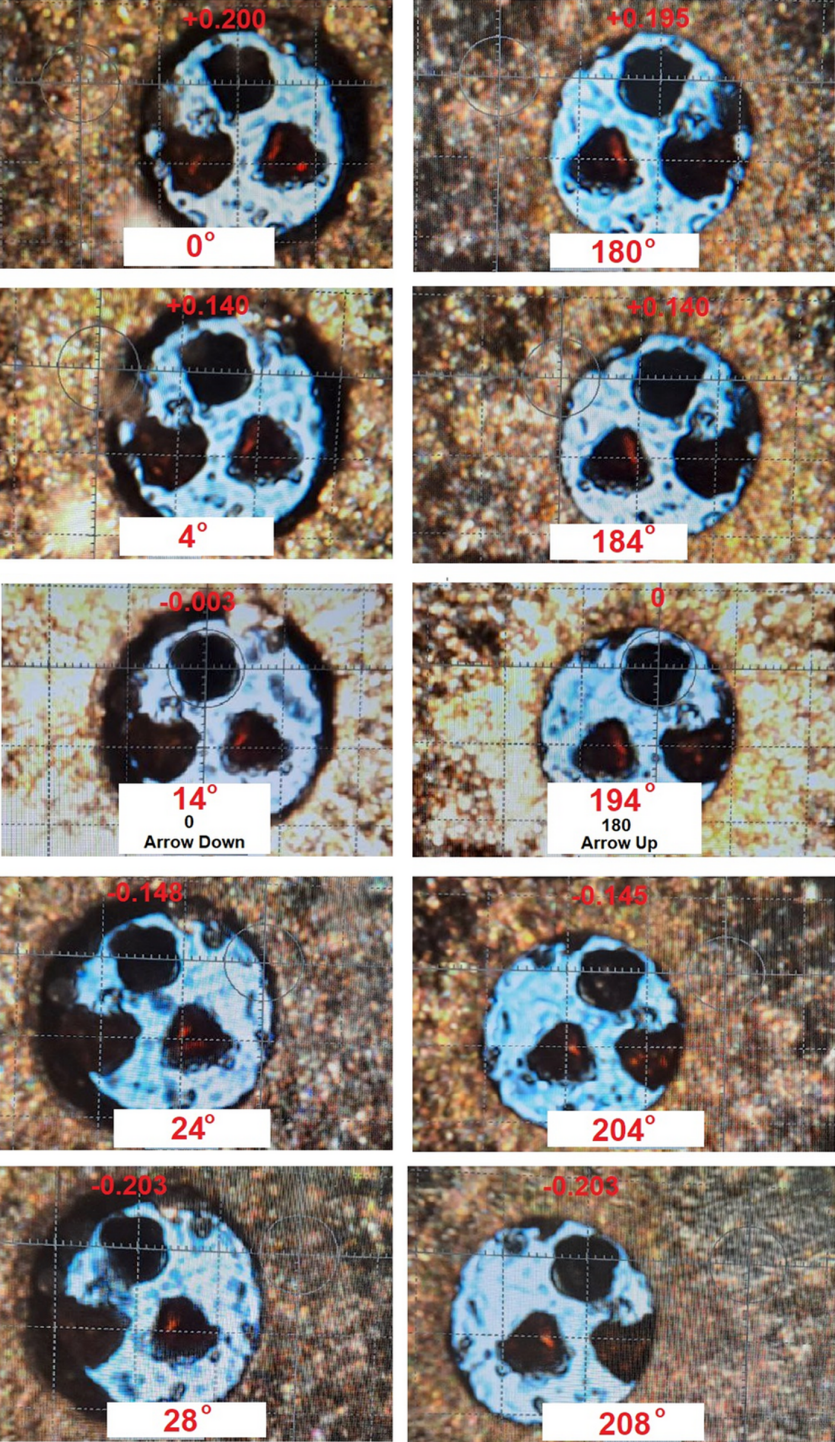
Apparent crystal position images of a well centered crystal in the DAC, obtained using the new and improved method with DAC tilts of φ ±10 and 14°. The images display the corresponding φ angles on the diffractometer and indicate the crystal displacement in millimetres. Three different crystals are simultaneously placed in the working chamber of the DAC; only the top one is used for centering.

Adjusting the DAC in the direction of the microscope to minimize these deviations is crucial for accurate centering. Crystal and DAC alignment at higher tilt angles can further enhance precision. While angles of 10–14° are generally optimal and good enough for centering, larger angles >18–20° can compromise the alignment due to defocusing effects and image distortion caused by the longer light path through the diamond. Conversely, smaller angles (4–6°) limit accuracy. The images in Fig. 6 ▸ depict apparent crystal positions of a well centered crystal in the DAC, obtained using the improved method with DAC tilts of ±10 and ±14°. The images display the corresponding φ angles on the diffractometer and indicate the crystal displacement in millimetres. Three different crystals are simultaneously placed in the working chamber of the DAC; only the top one is used for centering. The data in Table 2 ▸ summarize the crystal center position values taken from Fig. 6 ▸ after tilting the DAC by φ angles of ±10 and 14°. As can be seen from the data presented, the values of the deviations of the crystal centers in pairs of angles coincide within 0.005 mm, demonstrating perfect centering. Analysis of the absolute values of the crystal center positions at symmetric angles in the positive and negative tilt angles reveals maximum deviations of 0.008 mm. These deviations are likely attributable to a combination of factors, including the crystal’s imperfect shape, the accuracy of the initial alignment in step (1) and the influence of the additional PTM layer. A noteworthy observation is that identical crystal center positions are observed at the same tilt angles in the specific DAC. For example, at a tilt angle of −10° for the DAC, the crystal remains precisely centered when the apparent crystal center is at the position 0.140 mm, and for a tilt angle +14° the center of the crystal is at −0.203 mm (Tables 1 ▸ and 2 ▸). This suggests that the crystal position in a DAC with predetermined parameters (depending on the DAC thickness) can be accurately aligned using only one tilt angle, even without the need for a 180° rotation. Although this simplified method significantly enhances the applicability of crystal alignment using DAC tilt, especially when a full 180° rotation is not possible, it may compromise the accuracy of crystal alignment, especially for irregularly shaped crystals.

Averaging multiple measurements of the apparent crystal center position, including both positive and negative tilt angles for the DAC, significantly enhances the accuracy of determining the crystal center and its deviation from the goniometer center. This approach can achieve an unprecedented level of precision, with crystal position determination accurate to within approximately 0.005 mm, which corresponds to a displacement of the crystal in the direction of the microscope of about 0.01 mm after the centering using this method.

Employing a microfocus radiation source enables sequential diffraction experiments on multiple crystals/compounds within the same DAC at a fixed pressure. This significantly streamlines the high-pressure diffraction process by requiring complete centering only once. In this approach, only the upper crystal is centered (Fig. 6 ▸), followed by a simple lateral displacement of subsequent crystals towards the goniometer center without the need for full recentering. This improved optical centering method typically yields datasets collected from the samples in the DAC with *R*_int_/*R*_σ_ values below 0.05 for well diffracting crystals, enabling excellent refinement parameters (Rusanov *et al.*, 2024*b* ▸). This demonstrates the effectiveness of the method in achieving precise crystal alignment. In contrast, optical centering relying solely on microscope focus often results in higher *R*_int_/*R*_σ_ values (above 0.15–0.2) and poorer refinement parameters, which is the reason for its use only as an initial centering method. The method also speeds up the alignment process by eliminating the need for multiple data acquisition cycles with indexing and unit-cell parameter refinement during crystal alignment or gasket image inspection without a beamstop under reduced power (for safety reasons). Although this method offers exceptional speed and accuracy, its applicability may be limited by the design of the DAC. In particular, DACs with beryllium plates over anvils containing pinholes may obscure crystal visibility at angles of about 10°. Ensuring accurate parallel alignment of the DACs is also critical to the effectiveness of this method when rotating the DAC for 180°, but it is not necessary for known DAC geometry parameters as noted above.

Finally, we briefly compare our crystal centering method in the DAC with other methods that are used for high-pressure experiments at synchrotrons. Diffractometric centering methods are complex and time-consuming techniques that require specialized software modules for calculations. These methods involve iterative processes of data collection and analysis, making them less efficient compared with other approaches. Currently, crystal alignment at synchrotrons relies on older, relatively fast methods described in the *Introduction*[Sec sec1]. Motorized six-circle diffractometers enable effective scanning of the gasket/chamber absorption profile (Zhang *et al.*, 2017 ▸) for the gasket shadowing method. This technique also aids in directly and accurately determining the position and centering of strongly absorbing samples (Lotti *et al.*, 2020 ▸); however, this technique is limited to weakly absorbing crystals. Although placing an additional small but strongly absorbing marker in the chamber can help (Glazyrin *et al.*, 2022 ▸), it introduces additional diffraction and scattering noise, and the alignment method remains indirect. Many modern high-pressure beamlines, particularly those at fourth-generation synchrotron sources such as the EMA beamline at SIRIUS and the upgraded ID-27 beamline at ESRF, utilize focused beams with sizes less than 1 µm. However, some experiments, particularly those involving samples with weak scattering power (*e.g.* organic crystals) still employ wide beams. Such high-pressure diffraction experiments can be performed on synchrotron beamlines using a wide beam and standard optical centering methods using microscope focusing (Boer *et al.*, 2023 ▸). This approach ensures that the misaligned crystal remains within the beam during data collection, and a wide beam can improve diffraction intensity from samples with weak scattering power. However, this can also lead to degraded diffraction data due to increased powder diffraction and fluorescence noise from the gasket material. Our proposed method offers a significant advantage by relying solely on the visible image of the sample. This eliminates the need for scattering or absorption information and allows for the effective use of microfocus sources. Precise optical centering enables the simultaneous placement of multiple samples in the DAC and their independent data collection, regardless of absorption, scattering power, color or shape.

## 4. Conclusions

A new precise optical method of crystal centering by tilting of the DAC was introduced and used for crystal centering. The novelty of our approach lies in its reliance on purely optical methods, rather than diffraction or absorption techniques, to determine the sample position within the DAC. The primary advantages of this method include high accuracy, simplicity, rapid centering and immediate visual feedback. Even minor crystal displacements along the microscope axis result in noticeable changes to the crystal image displacement. For precise crystal centering, a tilt angle in the range 10–14° can be used, which ensures good crystal centering accuracy. The method is suitable for instruments with a Boehler–Almax DAC design. This method provides significant safety advantages when working with X-ray and synchrotron sources. It eliminates the need to remove the beam stop and expose the DAC to the primary beam to check the position of the gasket hole or the internal highly absorbing very small crystal for alignment with the detector, reducing the risk of damage to the instrument and potential harm to the user.

We have extensively tested this method in numerous high-pressure diffraction experiments, including those reported in our recent publications. Through these trials, we have confirmed its exceptional efficiency and accuracy, as shown by the consistently low *R*_int_ values and high-quality data refinement parameters achieved. Given the limited availability and high cost of synchrotron beam time, this precise and fast centering procedure is essential to minimize the experimental time and optimize data collection in diffraction experiments.

## Figures and Tables

**Table 1:** Apparent crystal center positions after DAC tilting at φ angles of ±6, 8, 10 and 14° for a crystal in the DAC centered using the standard optical method by focusing the microscope The crystal center position is determined by calculating the average of the apparent crystal positions observed on its the left and right sides. A crystal deviation to the left of the microscope center is considered negative, and a deviation to the right is considered positive.

φ (°) (DAC tilt, °)	Crystal center position (mm)	φ (°) (DAC tilt, °)	Crystal center position (mm)
0 (−14)	+0.182	180 (−14)	+0.220
4 (−10)	+0.122	184 (−10)	+0.157
6 (−8)	+0.097	186 (−8)	+0.127
8 (−6)	+0.075	188 (−6)	+0.097
14 (0)	0	194 (0)	0
20 (+6)	−0.075	200 (+6)	−0.095
22 (+8)	−0.097	202 (+8)	−0.125
24 (+10)	−0.125	204 (+10)	−0.155
28 (+14)	−0.180	208 (+14)	−0.225

**Table 2:** Apparent crystal center positions after DAC tilting at φ angles of ±10 and 14° for a well centered crystal in the DAC using the new and improved optical method The crystal center position is determined by calculating the average of the apparent crystal positions observed on its left and right sides. A crystal deviation to the left of the microscope center is considered negative and a deviation to the right is considered positive.

φ (°) (DAC tilt, °)	Crystal center position (mm)	φ (°) (DAC tilt, °)	Crystal center position (mm)
0 (−14)	+0.200	180 (−14)	+0.195
4 (−10)	+0.140	184 (−10)	+0.140
14 (0)	−0.003	194 (0)	0.000
24 (+10)	−0.148	204 (+10)	−0.145
28 (+14)	−0.203	208 (+14)	−0.203
